# Simultaneous coronary artery bypass grafting and carotid endarterectomy can be performed with low mortality rates

**DOI:** 10.1186/1749-8090-10-S1-A301

**Published:** 2015-12-16

**Authors:** Ebuzer Aydin, Yucel Ozen, Sabit Sarikaya, Davut Cekmecelioglu, Ismail Yukseltan

**Affiliations:** 1 Kartal Kosuyolu Yuksek Ihtisas Research and Training Hospital, Istanbul, Turkey; 2 Taksim German Hospital, Istanbul, Turkey

## Background/Introduction

There remains a controversy on the best approach for patients with concomitant carotid and coronary artery disease.

## Aims/Objectives

In this study, we report our experience with simultaneous carotid endarterectomy (CEA) and coronary artery bypass graft (CABG) surgery in our clinic in the light of the literature data.

## Method

Between January 1996 and January 2009, a total of 110 patients (86 males, 24 females; mean age: 65.11 ± 7.81 years; range, 44 to 85 years) who were admitted to hospital, Cardiovascular Surgery Clinic were retrospectively analyzed. All patients underwent simultaneous CEA and CABG. Demographic characteristics of the patients and a history of previous myocardial infarction (MI), hypertension, diabetes mellitus, hyperlipidemia, peripheral arterial disease, and smoking were recorded.

## Results

One patient (0.9%) with major stroke died due to ventricular fibrillation. Perioperative neurological complications were observed in seven patients (6%). Complications were persistent in two patients. Four patients (3%) had postoperative major stroke, whereas three patients (2%) had transient hemiparesis. No perioperative myocardial infarction was observed.

## Discussion/Conclusion

Simultaneous CEA and CABG can be performed with low mortality and morbidity.

